# Methodology for building a geographical accessibility health index throughout metropolitan France

**DOI:** 10.1371/journal.pone.0221417

**Published:** 2019-08-22

**Authors:** Ludivine Launay, Fabien Guillot, David Gaillard, Mohand Medjkane, Thierry Saint-Gérand, Guy Launoy, Olivier Dejardin

**Affiliations:** 1 U1086 INSERM "Anticipe", Caen, France; 2 Centre François Baclesse, Caen, France; 3 University of Caen Normandie, Caen, France; 4 UMR 6266 CNRS IDEES, Caen, Rouen, Le Havre, France; 5 Research department, University Hospital of Caen, Caen cedex, France; University of Essex, UNITED KINGDOM

## Abstract

Spatial accessibility to health services is a key factor in terms of public health. Even though some tools are available, establishing accessibility criteria applicable from one geographic scale to another remains difficult. Therefore, we propose a method for creating a health accessibility index applicable on a large geographic scale, based on a methodology that overcomes the limitations of political-administrative divisions and which allows a multi-scalar approach to be implemented. The index highlights, on a national scale, areas of cumulative health disadvantages. This index of accessibility to health care combines accessibility and availability and can be adapted to many geographical scales. As accessibility can be understood in various dimensions, a score could be calculated for various fields such as education and culture. The index can help policymakers to identify under-endowed areas and find optimal locations. In terms of public health, it may be used to understand the mechanisms underlying geographic health disparities.

## Introduction

The spatial accessibility to health services is a key factor in terms of public health. Its assessment is based on different concepts and measures, the distance to a health professional, the nearest hospital or the closest reference treatment centre being some of the most frequently used. The greater the distance, the lower the access to a health facility. For example, it has been found that patients diagnosed with cancer and living far from a reference care centre are less likely to receive certain types of treatment and therefore have lower chances of survival [1–3]. The implementation of a public health policy requires the use of specific tools on a national scale. While various tools exist to assess geographic units such as regions, inter-area comparisons are either difficult or impossible to make, so designing a relevant national public health policy is very challenging. The same applies to research fields where comparisons between studies involves the use of the same measurement tool.

A distinction should be made between potential for access and effective remedy [4]. The development of measurement tools has been the subject of many studies in recent decades and various indicators are available for potential spatial accessibility. Medical density (supply/demand ratio) and/or medical service (demand/supply ratio) were used initially. These have the advantage of being easy to calculate and comprehensible for decision-makers, but they do not allow for border crossing and require equivalent accessibility for all professionals in the same area. The distance to the nearest facilities or access time are also considered. While the notion of distance allows for border crossing, it does not include the entire available supply, so it is an unreliable indicator of actual availability, particularly in urban areas. Gravity models, on the other hand, seek to represent the interaction between the population and the entire supply within a reasonable distance [4]. For a given spatial unit (often the administrative unit centroid), the idea is to calculate the sum of the ratios between the supply (often the number of professionals) situated at a certain distance, and the distance to the centroid. Therefore, they make it possible to take both the accessibility and availability of the resource into account. However, since this measure is not very intuitive in comparison to a density or a strict distance and does not take the demand on the supply itself into account, variations of these models have been proposed. Some of them include the demand for supply (2SFCA) [5–10], while others incorporate the diversity of transportation modes [11,12], travel time considerations using the “enhanced two-step floating catchment area” model or the E2SFCA model [13], and competition between suppliers (3SFCA) [14]. The Two-Step Floating Catchment Area (2SFCA) method has been used and improved in Australia [15,16], in Colombia [17], in France to construct the notion of localized potential accessibility (LPA) [18,19]. While these models make it possible to integrate supply and demand within the same measure, they do not to take the “distance decay” into account [13], i.e. the differentiated use of resources depending on whether they are located close to the applicant or the catchment area borders. Furthermore, there are different ways to consider both supply and demand, such as the number of professionals, the number of full-time equivalent employees, etc.

These modifications were made in order to calculate the LPA. However, the main limitation of these measures is the use of a fixed scale (i.e. the municipality), because it is important to have a transposable measure of accessibility from one geographic scale to another. Moreover, they only consider one medical professional in a single value, even though the notion of accessibility to health care concerns several disciplines that should be considered together. This is what has been done in Great Britain with a multidimensional small area index named "Access to Healthy Assets and Hazards" index. It includes three domains of accessibility: health services, retail outlets, and environment quality [20]. In this paper, we propose a method for creating a health accessibility index, named the SCALe (Spatial aCcessibility multiscALar) index, based on distance and not on medical density on a large geographic scale that could also be adapted to smaller ones. To do so, we use a construction methodology that overcomes the limitations of political-administrative divisions and allows a multi-scalar approach to be adopted. The methodology is applied to data concerning France.

## Materials & methods

### Materials

Instead of using the centroid of an administrative unit, we used residential buildings. Owing to the lack of data on this scale, we first estimated the number of inhabitants within this type of spatial unit. Thus, the first phase of this project was dedicated to preparing data as described in S1 File and the second to creating the index itself.

The equipment for metropolitan France was obtained from the Permanent Facilities Database (BPE) of 2013 provided by INSEE in its geolocated version. The BPE includes data from three directories: ADELI (Automated Lists of Health Professionals), RPPS (Shared Directory of Health Professionals) and FINESS (National Register of Health and Social Establishments). For the data from the first two directories, staff members are included in the BPE based on professional criteria, occupational status and sector of activity (private practice only). After excluding non-located equipment (4.3%), the data included 264,416 items of equipment as presented in S1 Table.

### Methods

The equipment was divided into categories and mainly concerned the provision of primary health care. Therefore, each category included different types of equipment (primary, secondary and tertiary), as defined by INSEE, depending on the frequency of use of these services by the population. The primary range includes equipment related to primary care (general practitioners, physiotherapists, nurses, pharmacists, dentists). The secondary range contains less frequently used, yet relatively numerous, types of equipment (for example, speech therapy, pedicure, radiologist, psychologist, analysis laboratory and medical biology, ambulance services, accommodation for the elderly, which are not included in this index). Finally, the tertiary range is comprised of rare, more specialized or larger types of equipment destined for medical obstetricians and gynaecologists, maternity wards, paediatric specialists, ophthalmologists, short-stay care services and accident and emergency departments.

**Fig 1:**
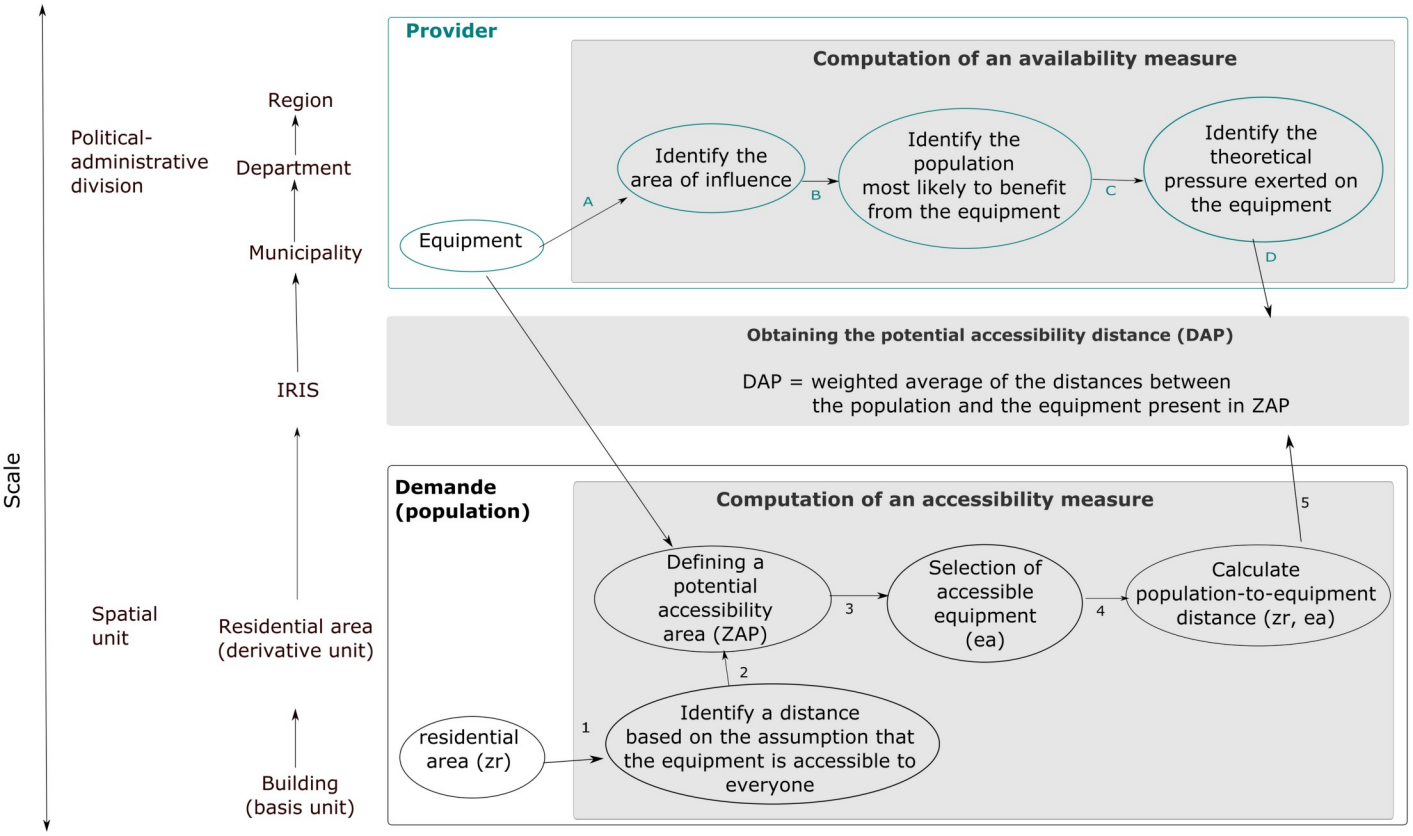
Construction of value per category. (*) average population—median population for each scale: region = 2 852 965–2 132 882; department = 653 804–534 139; municipality = 1 714–428; IRIS = 1 250–727, 2 372–2 347 using only IRIS different from municipality; residential area = 23–1.

The construction of a value for each category was based on the following diagram (Fig 1). In defining the accessibility of a resource, we took both its availability (the pressure exerted by users) and its spatial accessibility into account. Access to equipment was determined by a demand-oriented approach using the supply-weighted average distance to equipment.

### Computation of an accessibility measure

**Fig 2:**
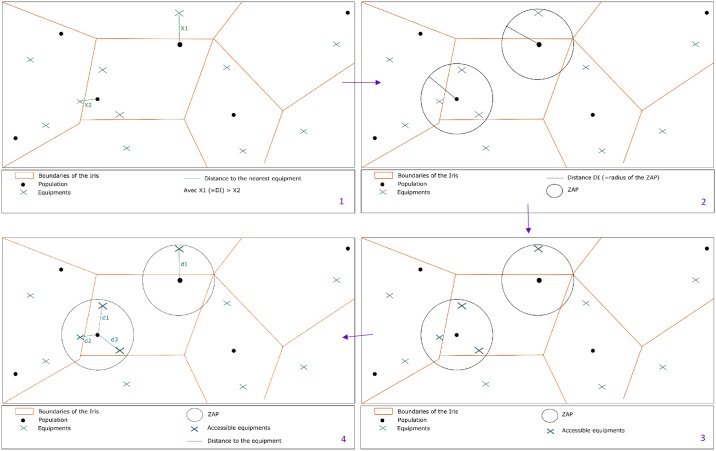
Calculation of potential accessibility distance.

In this demand-oriented approach, geographic accessibility was measured by the distance (distance between the residential area and the equipment) that the population had to travel in order to access a service. Thus, within the same spatial unit, the distance covered by an individual will depend on the supply available in a neighbourhood close to their dwelling place and implying a choice on their part (Fig 2). Long distances were associated with areas of low accessibility.

Based on the assumption that the equipment is accessible to everyone, the search radius of this neighbourhood area was defined by the highest Euclidean distance between the population of a same IRIS and the nearest equipment. This distance, named DI (1), allowed us to define a potential accessibility area (ZAP) that would ensure access to the equipment by the population, at a distance not exceeding DI (2). The notion of ZAP was used to select the accessible equipment devices (3) that served to calculate the potential accessibility distance (DAP). The DAP was defined as the weighted average of the distances between the population and the equipment present in a ZAP (4).

Using a Voronoi diagram, we weighted the population-to-equipment distance to integrate availability in order to express a pressure rate (5-D).

### Considering the provider: Computation of an availability measure

The area of influence of every item of equipment was established by a Voronoi diagram (A) to obtain a theoretical mesh of space. The limits of the Voronoi diagram are determined by the distance to the point of origin [21]. The method is based on two theoretical assumptions: first, that the borders cancel out the competitive effect of equipment devices between one another; second, that the behaviour of the population fits the theoretical optimization of the distance allowing for the construction of these borders. These mosaics define the population most likely to benefit from the equipment. For example, for medical offices, the number of professionals present was taken into account (B).

The population theoretically served was related to:

- the population of the “Etablissements Publics de Coopération Intercommunale” (EPCI) for primary equipment,
- the regional population for the range of tertiary equipment,

The figures took into consideration the population in need of the equipment (for example, the number of residents per gynaecologist). We were thus able to define the theoretical pressure exerted on the equipment (C).

Following this, the potential accessibility distances were transformed by the Box-Cox method [22] so that each of them followed a normal law according to the formula given by Proc TRANSREG using SAS^®^ (1):

$$t\_dap=\frac{{dap}^{\lambda }-1}{\lambda },\;\lambda \neq 0.$$ (1)

t_dap=ln(dap),λ=0

With λ = 0.20 for physiotherapists, general practitioners; 0.22 for dentists; 0.21 for pharmacists; 0.18 for nurses; 0.12 for paediatricians and for specialists in gynaecology and obstetrics maternity wards; 0.10 for short-stay care services; 0.13 for ophthalmologists; 0.04 for accident and emergency departments). Potential accessibility distances equal to zero were replaced by 0.0000001.

These new variables were standardized and then combined to obtain an overall health value for the residential area, the Spatial aCcessibility multiscALar index (SCALe index). The domain value was obtained by using the weighted sum of transformed potential accessibility distances. The weighting took the equipment range into account. The lack of access to a nearby facility such as a general practitioner’s office may be more detrimental to daily life than access to a tertiary range of equipment, such as that found in a hospital, which is less frequently used. General practitioners are the first health professionals that the population consults, so the number of each item of equipment was divided by the number of general practitioners to obtain the frequency of the equipment compared to the number of general practitioners’ offices. This ratio was the weight applied to transformed potential accessibility distances. These weights were: 1.225 for nurse (= 72,801/ 59,411), 1 for general practitioners, 0.99 for physiotherapists, 0.598 for dentists, 0.393 for pharmacists, 0.085 for specialists in gynaecology and obstetrics maternity wards, 0.081 for ophthalmologists, 0.044 for paediatricians, 0.025 for short-stay care services and 0.010 for accident and emergency departments (= 613/59,411). Thus, a high value of the potential accessibility distance for general practitioners and nurses penalizes the value of the score more than other equipment.

Let E be the set of health equipment

$${index}_{health}\;=\;\sum_{e\;\in E}W_{e}{DAP}_{e}$$

Therefore, the low values of the index are representative of a very high accessibility to the facilities of the field concerned. In addition, an interpolation of these distances was carried out using the Inverse Distance Weighted method (a second-degree polynomial taking the 12 closest neighbours into account) in order to cover the entire national territory. The same method was used for the health index. All the mapped variables were divided according to deciles with the darkest colour indicating the greatest inaccessibility. The scale changes involved averaging the index values to the geographic unit considered at IRIS level. The choices made for the cartographic representation were conserved. The construction of the accessibility index was programmed in Python and executed with ArcGIS^®^ 10.4. The data description was carried out with SAS^®^ 9.4 software. The diagrams were made with Inkscape software version 0.91.

### Sensitivity analysis

Since distances are the cornerstone of the calculation of this index, we performed a sensitivity analysis on different distance calculations. Analyses were done with Minkowski distance (with an exponent of about 1.25) and road network distance radius for the Lyon conurbation [23]. The three versions of DAP (Euclidian, Minkowski, Road network) were transformed and the accessibility index was calculated with each version as explained in S2 File. Road network distance was calculated using NAVSTREETS^©^ V6.3 and Network Analysis for ArcGIS Desktop.

## Results

**Fig 3:**
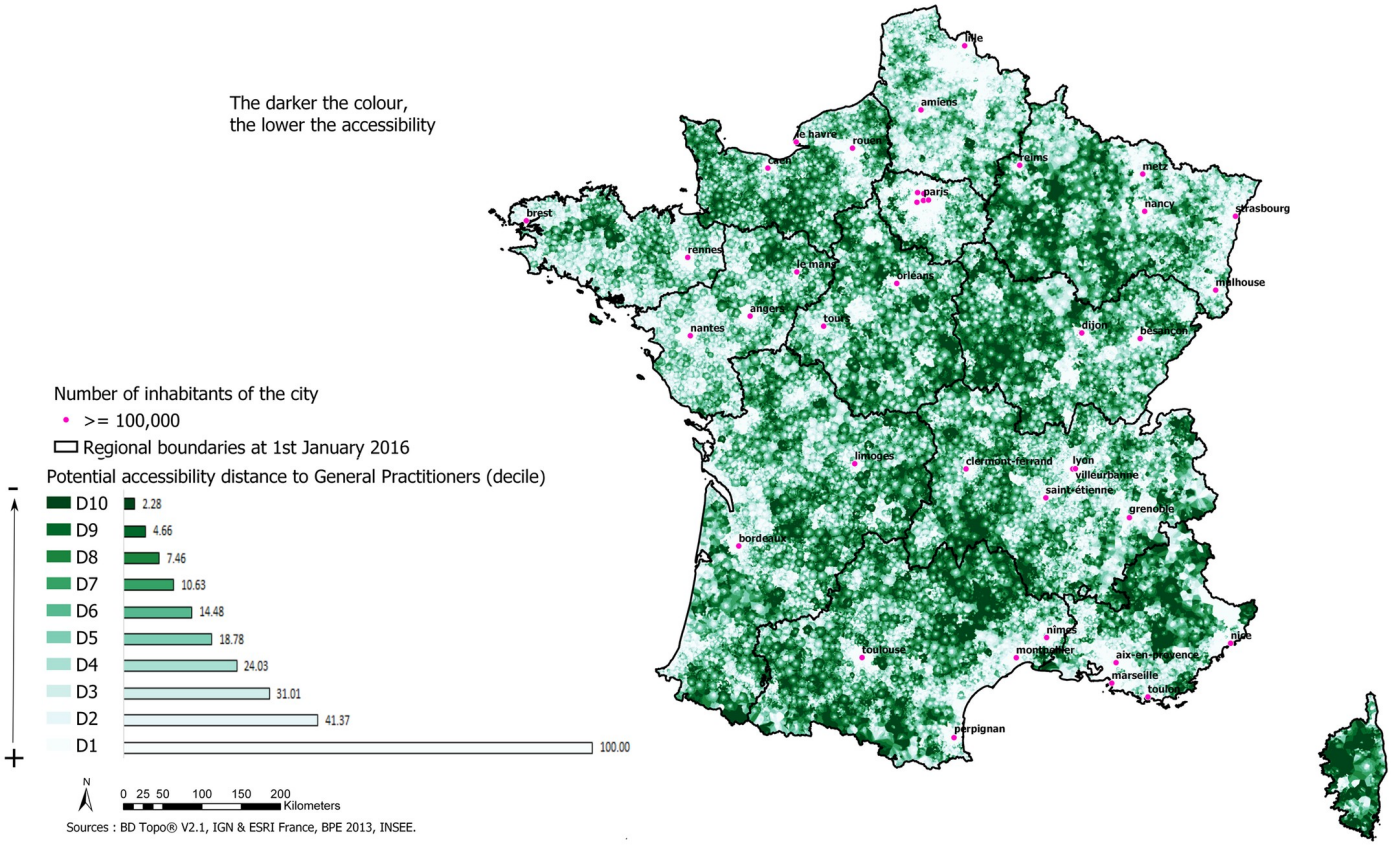
Potential accessibility distance to primary equipment (example for GPs).

With regard to local amenities, many areas of the territory were highly accessible (Fig 3).

The accessibility index was distributed as follows (Table 1):

**Table 1:** Distribution of accessibility index.

	Points	Total Population			Men	Woman	Age (year)					
Decile	Values/limits	n	%	cumulative percentage	n	n	00–19	Column percent	20–64	Column percent	≥65	Column percent
10	4.53; 22.18	1,292,002	2.06	2.06	650,764	641,239	297,147	1.94	734,172	2.01	260,683	2.45
9	3.13; 4.53	1,369,565	2.19	4.25	687,713	681,852	325,394	2.12	783,013	2.14	261,157	2.46
8	2.07; 3.13	1,489,699	2.38	6.63	746,363	743,336	360,453	2.35	852,108	2.33	277,138	2.61
7	1.13; 2.07	1,787,966	2.86	9.49	891,627	896,339	438,352	2.86	1,024,465	2.8	325,149	3.06
6	0.22; 1.13	2,018,713	3.22	12.71	1,004,692	1,014,021	499,839	3.26	1,160,214	3.17	358,660	3.37
5	-0.70; 0.22	2,443,284	3.9	16.61	1,211,186	1,232,098	606,252	3.95	1,404,700	3.83	432,331	4.06
4	-1.74; -0.70	2,975,175	4.75	21.36	1,470,778	1,504,398	741,190	4.83	1,710,333	4.66	523,652	4.92
3	-3.01; -1.74	4,218,005	6.73	28.09	2,066,554	2,151,451	1,036,657	6.76	2,403,625	6.55	777,723	7.3
2	-4.88; -3.01	6,785,828	10.82	38.91	3,307,908	3,477,920	1,672,789	10.9	3,880,995	10.58	1,232,044	11.56
1	-15.71; -4.88	38,340,773	61.13	100.04	18,333,495	20,007,277	9,377,724	61.07	22,752,806	61.99	6,210,243	58.27

The residential area with the worst accessibility concerned 6.6% of the metropolitan population. The population age structure of these areas also includes a high proportion of people over 65 years of age.

**Fig 4:**
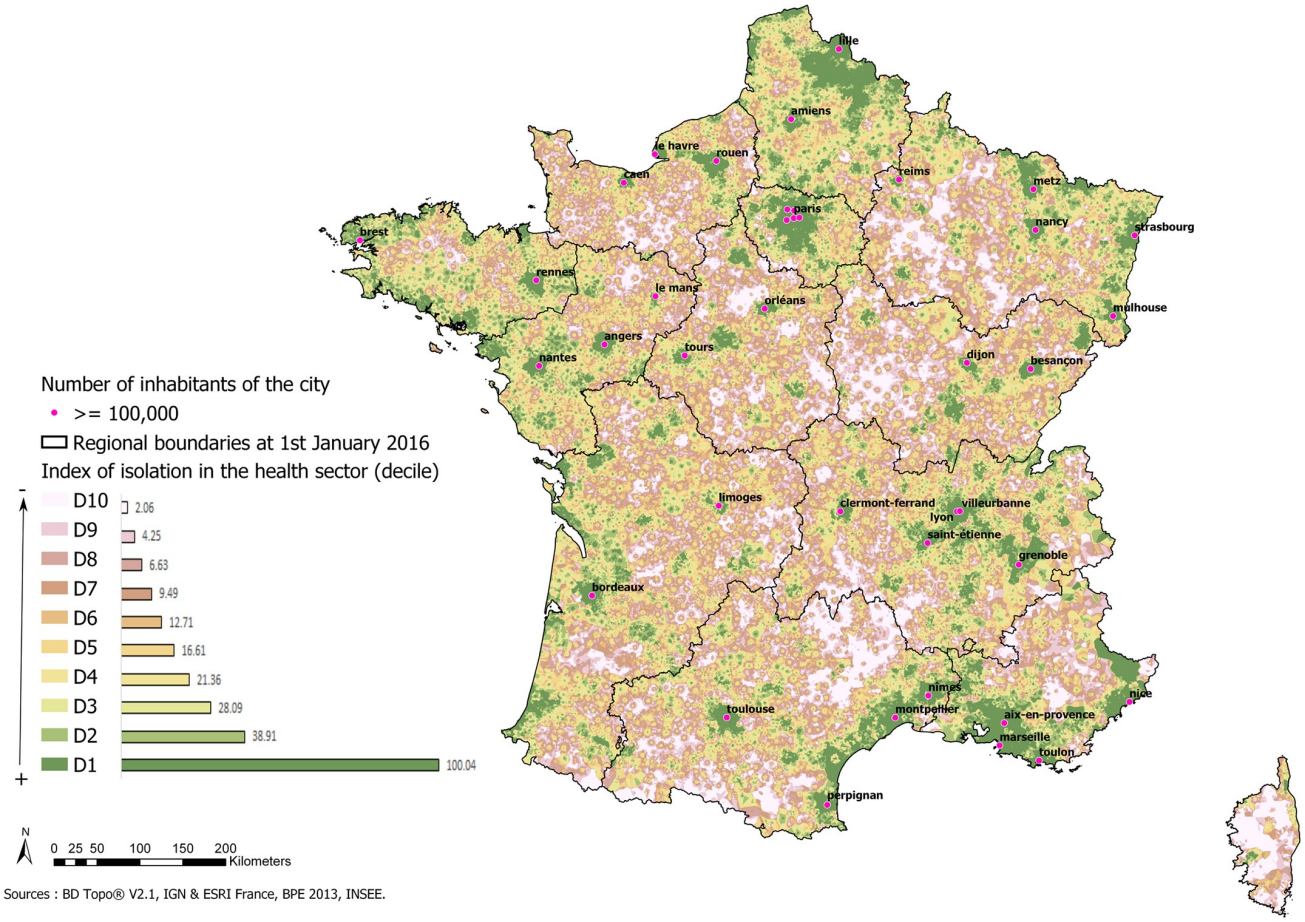
Index of accessibility to the health sector.

The cartographic representation of the accessibility index shows sections with high accessibility in the major urban centres. It indicates a very high level of inaccessibility in the western part of the Grand Est region, in Bourgogne-Franche-Comté, the north of Nouvelle-Aquitaine, the west of the Auvergne-Rhône-Alpes region and in Corsica (Fig 4).

At IRIS scale, the health accessibility index is distributed as follows (Table 2).

**Table 2:** Distribution of health accessibility index at IRIS level in metropolitan France.

Decile	Value	Population	%	Cumulative percentage	Men	Woman	00–19	20–64	>= 65
10	3.98–21.63	1,533,154	2.44	2.44	772,241	760,914	350,845	869,065	313,244
9	2.41–3.98	1,975,241	3.15	5.59	990,034	985,207	468,364	1,126,784	380,092
8	1.14–2.41	2,675,635	4.27	9.86	1,330,625	1,345,010	645,040	1,520,608	509,987
7	-0.05–1.14	3,692,452	5.89	15.75	1,820,870	1,871,582	893,990	2,089,203	709,259
6	-1.34–-0.05	4,531,036	7.22	22.97	2,233,269	2,297,768	1,119,627	2,581,546	829,863
5	-3.00–-1.35	5,790,956	9.23	32.20	2,847,770	2,943,186	1,443,309	3,325,206	1,022,441
4	-5.44–-3.00	7,884,779	12.57	44.77	3,855,819	4,028,960	1,983,073	4,559,039	1,342,666
3	-8.56–-5.44	10,928,799	17.42	62.20	5,294,999	5,633,801	2,789,505	6,368,831	1,770,463
2	-10.74–-8.56	11,901,329	18.98	81.17	5,676,526	6,224,803	2,989,608	7,027,617	1,884,104
1	-15.69–-10.75	11,807,626	18.83	100	5,548,928	6,258,698	2,672,436	7,238,532	1,896,659

**Fig 5:**
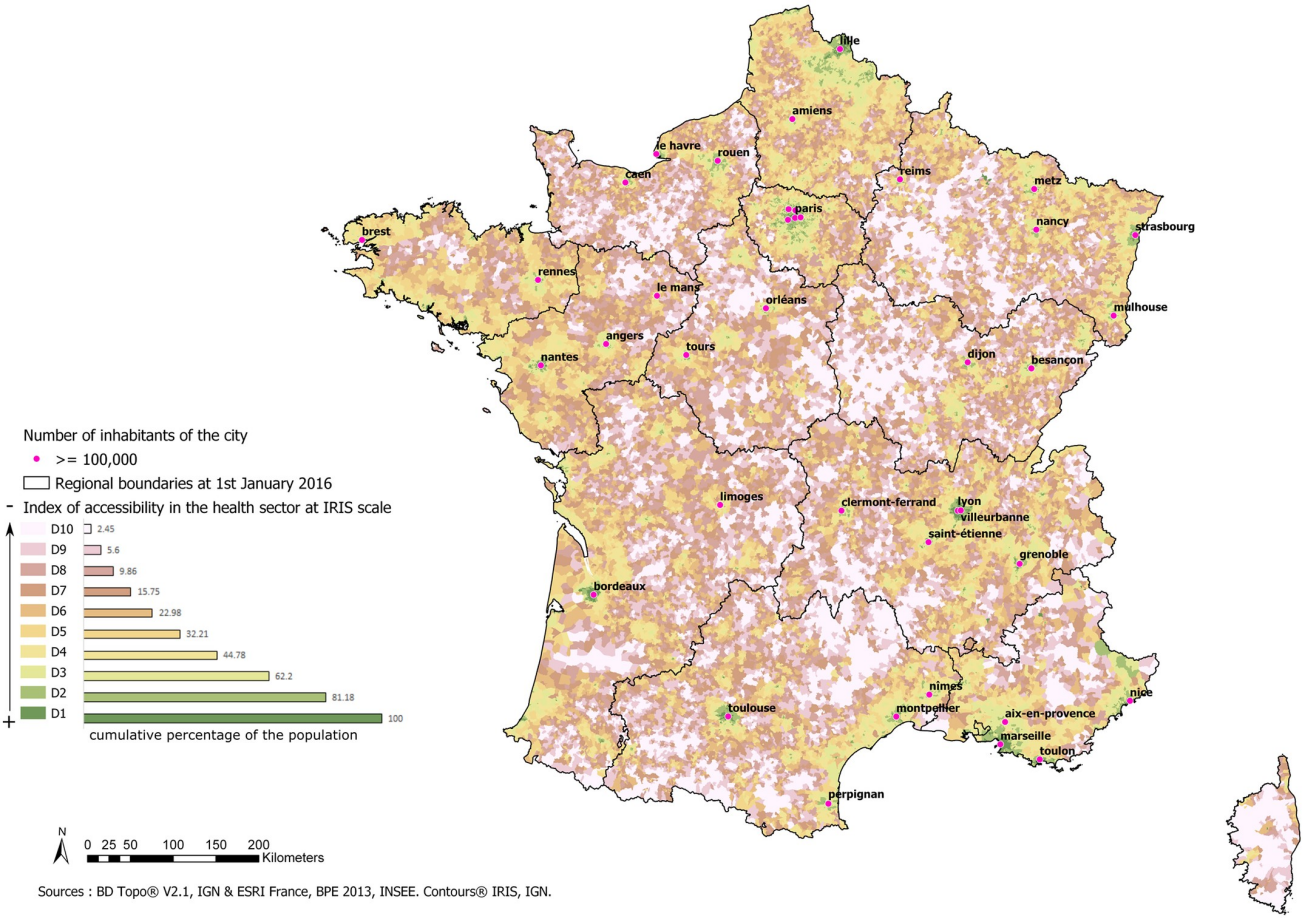
Health accessibility index at IRIS level in metropolitan France.

The IRIS with the worst accessibility concerned about 10% of the metropolitan population (Table 2). At IRIS level, the same most isolated areas are highlighted as at residential level. The least isolated areas are still located in major urban centres but are less spread out than previously (Fig 5).

### Sensitivity analysis

Using Euclidean or Minkowski distance on the Lyon conurbation, results highlight the greatest accessibility around Lyon, Villeurbanne, Rillieux la Pape, Oullins and Saint Priest. The lowest accessibility is around Quincieux, Lissieu (both located in the north) and Givors (in the south) (S2 File and S1 Fig). Results are quite similar with road network distance around Lyon concerning the greatest accessibility and Lissieu, Quincieux and Givors for the smallest accessibility. The index calculated with this distance highlights differences around Rillieu la Pape, Décines Charpieu and Saint Priest. The latter now has a small accessibility.

## Discussion

We constructed a multi-scalar health accessibility index (the Spatial aCcessibility multiscALar index) applicable nationwide and independent of political and administrative constraints. It may also serve to clarify choices regarding the location of public health facilities. Moreover, it allows the effects of changes in health care provision to be measured so that territorial disparities may be offset by appropriate regional planning. Combined with data on health indicators such as the incidence, lethality or mortality of a given pathology or the effects of screening, it may be used to measure the influence of geographical accessibility on the health status of the population. It can also be used as a monitoring tool within the framework of public policies targeting social inequalities.

Within mainland France, the index highlights areas of cumulative health disadvantages located especially in the west of the Grand Est region, Bourgogne-Franche-Comté, the north of Nouvelle-Aquitaine, the west of the Auvergne-Rhône-Alpes region and Corsica. Therefore, access to health care resources is insufficient in these areas. These zones comprising classes 8 to 10 represent about 6.6% of the population. The population structure of these areas also includes a high proportion of people over 65 years of age, so the problem is compounded by mobility difficulties and a high demand for care.

The study has several strengths. By taking residential buildings into account, it is possible to identify the precise location of the population more clearly than by using the centroid of an administrative unit. By locating the population in this way, it is possible to implement a multi-scalar approach with changes of scale that reveals not only arbitrary divisions such as administrative areas but also residential areas. This involves calculating distances between the population and a service supply. Another strength of this tool is that it allows users to visualize areas of accessibility. It shows the supply that is available close to people and does not impose an a priori choice on them. Furthermore, it circumvents the notion of administrative boundaries [4]. Therefore, it offers some of the advantages of gravity models based on the “Two-Step Floating Catchment Area” (2SFCA) [4].

Like any other national measure, the construction methodology for highly urbanized and less urbanized areas is the same. The index expresses only the spatial component of access to care. Unlike other accessibility indexes, it may be used for a particular area such as a highly urbanized area.

Geographical accessibility may be linked to socioeconomic disadvantages [24] and social deprivation. The index does not take into account the many forms of accessibility that may exist and hence their eventual cumulative disadvantages. In analysing the mechanisms producing socio-territorial health inequalities, deprivation can be either directly integrated into the index or measured outside of it by using another index [25–29] or a survey. One of the main limitations is that various forms of accessibility are taken into account in the same way. The same value may be obtained with different linear combinations of the factors in the index. However, various combinations may be used to develop a typology of areas. Using a variable radius to construct a potential accessibility zone (ZAP) is another limitation because of its influence on the score value, the latter being an average of distances. However, the residential areas on which this radius was built contain only one item of equipment per construction. Retaining the greatest distance to the nearest equipment at IRIS scale is also another limitation. Using municipality or regional scale, or even both, could have been as relevant depending on the type of equipment. However, this would have led to a significant increase in the accessibility of certain areas as well as to removing some disparities in access.

The availability of resources varies according to factors relating to time and space, such as frequency, pressure, period, periodicity, location and density. In the absence of data on indicators of availability, Voronoi mosaics were used to determine the area of influence of the equipment and calculate the theoretical pressure exerted on it. The method consisting in determining the area of influence by Voronoi mosaics is open to criticism, since it divides space into disjointed zones and gives the impression that there is no competition between services. Furthermore, it assumes that the behaviour of the population matches this optimization of distance. However, this does not reflect actual travel behaviour in space. Moreover, it does not provide information about the supply available. It presupposes that areas of influence of services located in the same place are identical, even though certain mechanisms such as the effect of a professional’s reputation lead to heterogeneity. In the absence of information on a national level regarding the real availability of supply, there is no other choice than to work by approximation. Moreover, the availability of resources also depends on the population age structure. This could have an impact on pressure exerted on equipment and could therefore influence accessibility. For some equipment, the pressure rate depends only on the population considered (i.e. for gynaecologists, only pressure from the number of women was taken into account; for paediatricians, only people under 19 years were considered). Moreover, thanks to its high flexibility, the index could be adapted to specific contexts (compute only for some age classes, only for women etc). Nonetheless, the inclusion of population age structure could represent a future improvement for the present index.

Finally, the use of the Euclidean distance to calculate the potential accessibility distance is another limitation of the index, because it is not representative of the distances actually travelled by the population, particularly in urban areas where signage has a major impact on the chosen itinerary. The Minkowski distance could be the best approximation of it (Euclidian distance under-estimates the real distance, Manhattan distance over-estimates it [23]) and should be integrated into the calculation of the potential accessibility distance as the distance by road. Furthermore, it is important to consider the distance to the network, i.e. the distance between users and the main lines of communication. This could not be done in this version of the index due to insufficient computation time. Nevertheless, a sensitivity analysis using Minkowski and road network distances was done for the Lyon conurbation. Euclidean and Minkowski distances were quite similar. With road network distance, with the notable exception of the north of the conurbation, results were broadly consistent with those obtained previously. Some of the differences observed were due to an insufficient number of points used for interpolation, which was responsible for the geometric configuration of the map.

Other limitations concern the data used as the BPE included only professionals in private practice. Moreover, some of the equipment present in the BPE was not located (from 0.1 to 11.5% depending on the equipment considered; average of 5.7%) or was wrongly located (1.3% of all facilities combined). This incompleteness of the data may have led to an overestimation of accessibility in some residential areas.

The methodology used to calculate the potential accessibility distance allows for border crossing and includes supply availability while taking the concept of distance into account. This has already been verified in various gravity models, including the 2SFCA [5–7]. This methodology has been improved to obtain the Localized Potential Accessibility (LPA) in France at the municipal level. These improvements concern the quantification of health care supply with the inclusion of full-time job equivalents, the quantification of the demand for health care based on the number of inhabitants standardized by their age structure and the incorporation of a weighting based on distance. The LPA expresses the density of professionals per municipality. Such administrative boundaries are often used to calculate potential accessibility indices and thus limit the scope of such tools. The multi-scalar dimension (i.e. each level for which existing geographic information can be used) combined with the possibility to suppress the effect of administrative boundaries were the cornerstones of the construction of the index presented here.

These results are in line with others obtained in France concerning general practitioners in private practice under 40 years of age in areas with very high accessibility. The trends in the least accessible areas in terms of local health care (general practitioners, private nurses, dentists, massage therapists and pharmacies) were also consistent with these findings, except for the PACA region [30]. The former regions of Champagne-Ardenne, Bourgogne and Corsica, which are considered to be areas affected by long waiting periods of access to health care, were also among those with poorer accessibility in this study [31]. The potential accessibility distance for nurses, ophthalmologists and paediatricians provided a cartographic representation that is consistent with those found in other studies, except for paediatricians in North Cotentin due to the non-exhaustive nature of the data mentioned above.

These results are also in accordance with those found in the state of Victoria in Southern Australia [7], in the United States [13], particularly in Florida [11], where accessibility was high in large cities.

## Conclusions

We propose an index of accessibility in health care that is on a detailed geographic scale but is adaptable to many geographical scales. To do so, we used an original construction methodology. The index may be used to study the impact of accessibility on different health indices measured on a geographical scale, such as incidence, lethality, mortality and screening.

Like the Index of Multiple Deprivation (IMD)[32] which is the British social deprivation index, the concept of accessibility can be understood in its various dimensions and constitutive modes, as well as the mutual links between them, i.e. social and cultural accessibility, socio-economic accessibility, geographic accessibility, disparities and inequalities in the spatial distribution of equipment and services. In this way, a score can be calculated for factors such as education and culture based on the methodology described here. The domains included in the accessibility index will therefore vary according to the objective of the study.

Accessibility is a phenomenon whose definition and intensity vary from one country to another depending on the type of space and the political context. For example, it could be calculated in Europe to allow for comparative studies across countries and particularly in the UK, where research has already shown interesting differences between health indicators and health care systems. In addition to being useful for a territorial diagnosis, this index can be compared with health data. It may be used for land use planning to identify under-endowed areas and find optimal locations. In terms of public health, it sheds light on the mechanisms underpinning geographic health disparities. Above all, it can be linked to health data in order to test the connection between geographic accessibility and the key indicators of a pathology.

## Glossary

Category: type of professionals or establishment

PAD (Potential Accessibility Distance): mean of distances to accessible facilities weighted by the pressure exerted on the equipment

DI: maximal Euclidean distance to the nearest equipment for residential areas belonging to the same IRIS

EPCI (Etablissements Publics de Coopération Intercommunale / Public Establishments of Intermunicipal Cooperation): groups of municipalities whose purpose is to develop “joint development projects within solidarity perimeters”. They are subject to common, homogeneous rules comparable to those of local authorities. These groups are of two types: the EPCIs with their own tax laws (e. g. the Urban, Agglomeration and Municipal Communities; the Syndicat d’ Agglomération Nouvelle; the Metropolis), and the EPCIs with no separate tax regime.

IRIS: (Îlots Regroupés pour l’Information Statistique): geographical entity created by INSEE in 2000 for the dissemination of the 1999 census, ensuring a homogeneous population within the same spatial unit. It divides all municipalities with more than 10,000 inhabitants and part of those with 5 to 10,000 inhabitants. Municipalities with fewer than 5,000 inhabitants make up a single IRIS.

Domain value: weighted sum of potential accessibility distances

PAZ (Potential Accessibility Area): constructed area based on the DI distance, which guarantees access to equipment for the residential area.

## Supporting information

S1 File Projection of the population on building right-of-way and height. (PDF)

S2 File Comparison of accessibility index based on Euclidean, Minkowski and road network distance. (PDF)

S1 Table Description of equipment. (PDF)

S1 Fig Sensitivity analysis—Accessibility index of health care (respectively with Euclidean, Minkowski and road network distances) in a major metropolis: The example of Lyon. (TIF)
