# Supplementary material for: Methodology for building a geographical accessibility health index throughout metropolitan France
Source: PLoS One. 2019 Aug 22;14(8):e0221417. doi: 10.1371/journal.pone.0221417 (PMC6705764; doi:10.1371/journal.pone.0221417)
Supplement: S1 File — (PDF) [file pone.0221417.s001.pdf]

S1 File. Projection of the population on building right-of-way and height.

The residential environment was extracted in several stages from the undifferentiated built environment of the BD Topo® V2.1 database of the IGN maps. First, an intersection between the undifferentiated built environment and activity surfaces allowed for the isolation of activity-related buildings (n=586,794). The latter were then removed from the undifferentiated frame by symmetric difference to obtain the residential frame (n=31,952,303).

The population size within these buildings was estimated using the 2010 census data provided by INSEE at the IRIS scale according to the geographical boundaries in effect on January 1st, 2012 (professionnels.ign.fr/contoursiris). The buildings were therefore previously divided up according to the outlines of this spatial unit (n=33,881,932).

The population size within the built environment was estimated proportionately to its surface area and number of floors. As the latter was not included in the BD Topo®, it was deduced from the height of the building as follows:

Let:

- h, the height of the building (in meters)
- e, the estimated number of floors
- s, the surface area of the building

From the height (in meters):

If h<5,

$$e = 1$$

If h≥5,

$$e = \frac{h}{2.5}$$

Let:

i, the elements of set I composed of IRIS: I={i_1, …, i_{ni}} with ni=50 195

b, the elements of set B composed of built environments: B={b_1, …, b_{nb}} with nb=33 881 932

For every built environment b included in IRIS i, the age group population a was estimated by:

$$p_{a,b\in i} = \frac{e_b \times s_b}{SE_i} \times p_{a,i}$$

Let:

- e_b, the estimated number of floors of the built environment b
- s_b, the surface area of the built environment b
- $SE_i = \sum_{b\in i} e_b \times s_b$
- $p_{a,\,i}$, the population at the IRIS scale for age bracket a

With a belonging to:

{'entire population', 'entire male population', 'entire female population', 'population aged between 00 and 19 years of age', 'population aged between 20 and 64', 'population aged 65+

years', 'male population aged between 00 and 19', 'male population aged between 20 and 64', 'male population aged 65+ years', 'female population aged between 00 and 19', 'female population aged between 20 and 64 years of age', 'female population aged 65+ years'}

Following this, residential areas were obtained by aggregating the residential built environment within a distance of 150 meters, without excluding any areas, while keeping all polygon holes and respecting the municipality boundaries (also derived from BD Topo® V2.1 of the IGN map). These areas were then delimited according to the IRIS outlines (Contours…IRIS® V 1.1 of IGN & ESRI France) to allow for scale changes. Nearly 2.8 million residential areas were thus created. The population size in residential areas amounted to the sum of the estimated inhabitants in the built environment considered, with the population located in the centroid of these built-up areas.
