# Supplementary material for: Methodology for building a geographical accessibility health index throughout metropolitan France
Source: PLoS One. 2019 Aug 22;14(8):e0221417. doi: 10.1371/journal.pone.0221417 (PMC6705764; doi:10.1371/journal.pone.0221417)
Supplement: S2 File — (PDF) [file pone.0221417.s002.pdf]

S2 File. Comparison of accessibility index based on Euclidean, Minkowski and Road Network Distance.

Minkowski Distance was calculated with an exponent of about 1.25 according to the formula:

$$D(x,y) = \sqrt[1.25]{\sum_{i=1}^{2} |x_i - y_i|^{1.25}}$$

Parameter λ used for Box-Cox transformation are those listed in table 1

S2 Table. Parameter λ used for Box-Cox transformation according to distance used to calculate the potential accessibility distance.

	Euclidean Distance	Minkowski Distance	Road Network Distance
Physiotherapists	0.05	0.05	0
General practitioners	0.05	0.05	0
Nurses	0.05	0.05	0
Pharmacists	0	0	-0.05
Dentists	0	0.05	-0.05
Short-stay care services	0.1	0.1	0.05
Paediatricians	0.05	0.1	0.1
Ophthalmologists	0.1	0.15	0.15
Specialists in gynaecology and obstetrics maternity wards	0.2	0.2	0.2
Emergency department	0.1	0.1	0.05

S1 Fig. Sensitivity analysis – Accessibility index of health care (respectively with Euclidean, Minkowski and Road Network Distances) in a major metropolis: the example of Lyon.

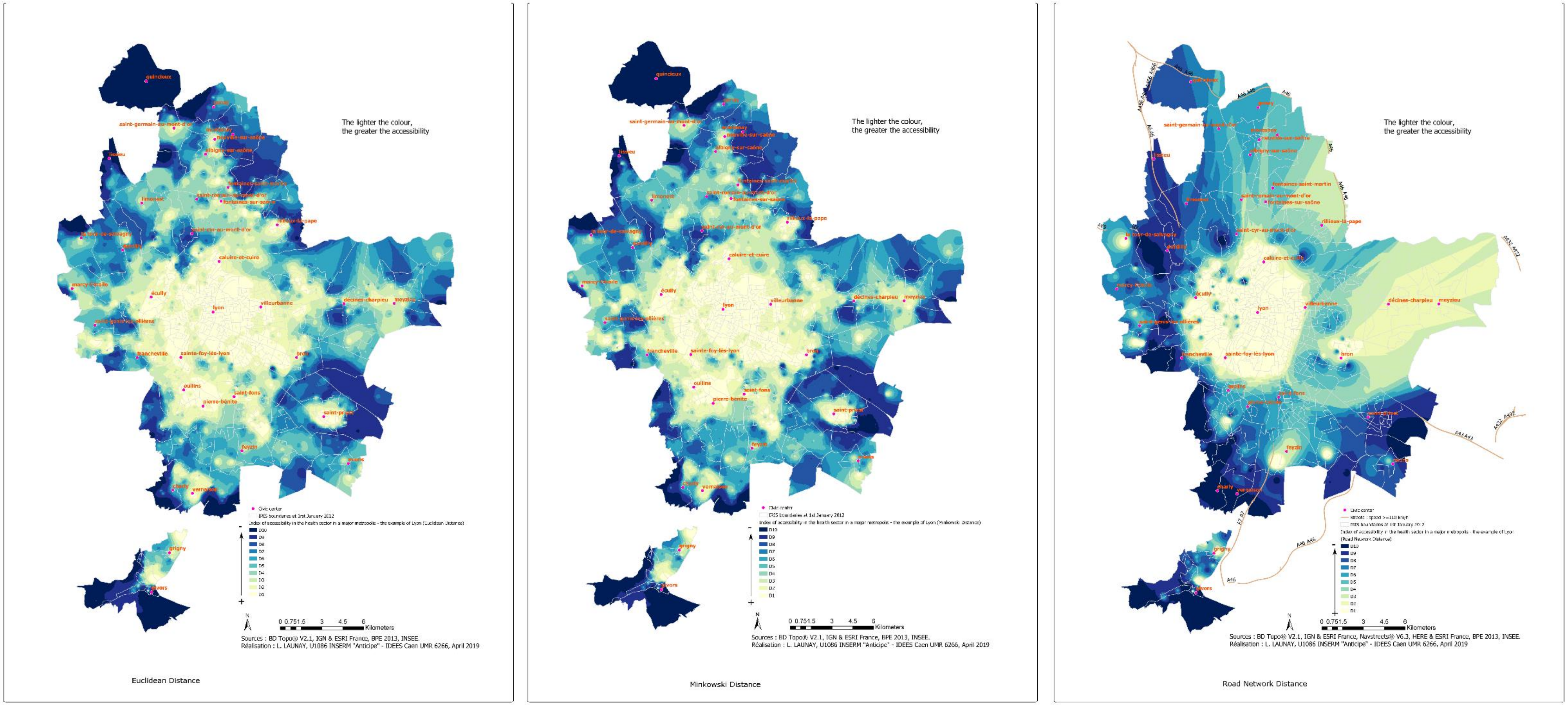

Annex: Sensitivity Analysis
