# Supplementary material for: Methodology for building a geographical accessibility health index throughout metropolitan France
Source: PLoS One. 2019 Aug 22;14(8):e0221417. doi: 10.1371/journal.pone.0221417 (PMC6705764; doi:10.1371/journal.pone.0221417)
Supplement: S1 Table — (PDF) [file pone.0221417.s003.pdf]

S1 Table. Description of equipment.

Region	Physio therapist	General practitioner	nurse	pharmacist	dentist	short-stay care service	emergency	gynecologists, maternity wards	pediatricians	Ophthalmo logist	Total
Auvergne-Rhône-Alpes	8 230 (14%)	7 291 (12.28%)	9 348 (12.85%)	2 803 (12%)	4 454 (12.55%)	176 (12.03%)	79 (12.89%)	568 (11.32%)	312 (11.93%)	572 (11.89%)	33 833
Bourgogne-Franche-Comté	1 981 (3.37%)	2 540 (4.28%)	2 650 (3.65%)	1 093 (4.68%)	1 235 (3.48%)	84 (5.74%)	29 (4.74%)	151 (3.01%)	71 (2.72%)	158 (3.29%)	9 992
Bretagne	3 185 (5.42%)	3 047 (5.13%)	4 611 (6.34%)	1 165 (4.99%)	1 883 (5.31%)	73 (4.99%)	30 (4.9%)	187 (3.73%)	112 (4.29%)	240 (4.99%)	14 533
Centre-Val de Loire	1 644 (2.8%)	2 087 (3.52%)	1 956 (2.69%)	905 (3.88%)	1 038 (2.93%)	54 (3.69%)	27 (4.41%)	162 (3.23%)	71 (2.72%)	146 (3.04%)	8 090
Corse	399 (0.68%)	292 (0.5%)	951 (1.31%)	148 (0.64%)	191 (0.54%)	11 (0.76%)	2 (0.33%)	15 (0.3%)	12 (0.46%)	23 (0.48%)	2 044
Grand-Est	4 080 (6.94%)	5 287 (8.9%)	5 133 (7.06%)	1 754 (7.51%)	3 248 (9.15%)	159 (10.87%)	61 (9.96%)	417 (8.31%)	255 (9.75%)	353 (7.34%)	20 747
Hauts-de-France	5 255 (8.94%)	5 473 (9.22%)	5 772 (7.93%)	2 226 (9.53%)	2 500 (7.04%)	128 (8.75%)	49 (8.00)	325 (6.48%)	148 (5.66%)	306 (6.37%)	22 182
Ile-de-France	10 012 (17.03%)	9 737 (16.39%)	7 039 (9.67%)	4 219 (18.06%)	7 293 (20.54%)	225 (15.37%)	91 (14.85%)	1 476 (29.4%)	786 (30.05%)	1 135 (23.6%)	42 013
Normandie	1 975 (3.36%)	2 829 (4.77%)	2 763 (3.8%)	1 072 (4.59%)	1 142 (3.22%)	80 (5.47%)	39 (6.37%)	178 (3.55%)	85 (3.25%)	204 (4.25%)	10 367
Nouvelle Aquitaine	5 559 (9.46%)	6 131 (10.32%)	8 137 (11.18%)	2 391 (10.24%)	3 353 (9.45%)	147 (10.05%)	67 (10.93%)	437 (8.71%)	172 (6.58%)	473 (9.84%)	26 867
Occitanie	7 115 (12.11%)	6 025 (10.15%)	11 426 (15.7%)	2 213 (9.47%)	3 690 (10.39%)	134 (9.16%)	67 (10.93%)	405 (8.07%)	227 (8.68%)	431 (8.96%)	31 733
Pays de la Loire	2 939 (5%)	3 192 (5.38%)	2 347 (3.23%)	1 256 (5.38%)	1 781 (5.02%)	76 (5.20%)	26 (3.75%)	188 (3.75%)	72 (2.76%)	294 (6.12%)	12 171
PACA	6 418 (10.92%)	5 480 (9.23%)	10 668 (14.66%)	2 126 (9.1%)	3 707 (10.44%)	117 (8.0%)	46 (10.22%)	513 (10.22%)	293 (11.21%)	476 (9.9%)	29 844
**Total**	**58 792**	**59 411**	**72 801**	**23 371**	**35 515**	**1 464**	**613**	**5022**	**2616**	**4 811**	**264 416**
