# Supplementary figures and images for: Methodology for building a geographical accessibility health index throughout metropolitan France
Source: PLoS One. 2019 Aug 22;14(8):e0221417. doi: 10.1371/journal.pone.0221417 (PMC6705764; doi:10.1371/journal.pone.0221417)

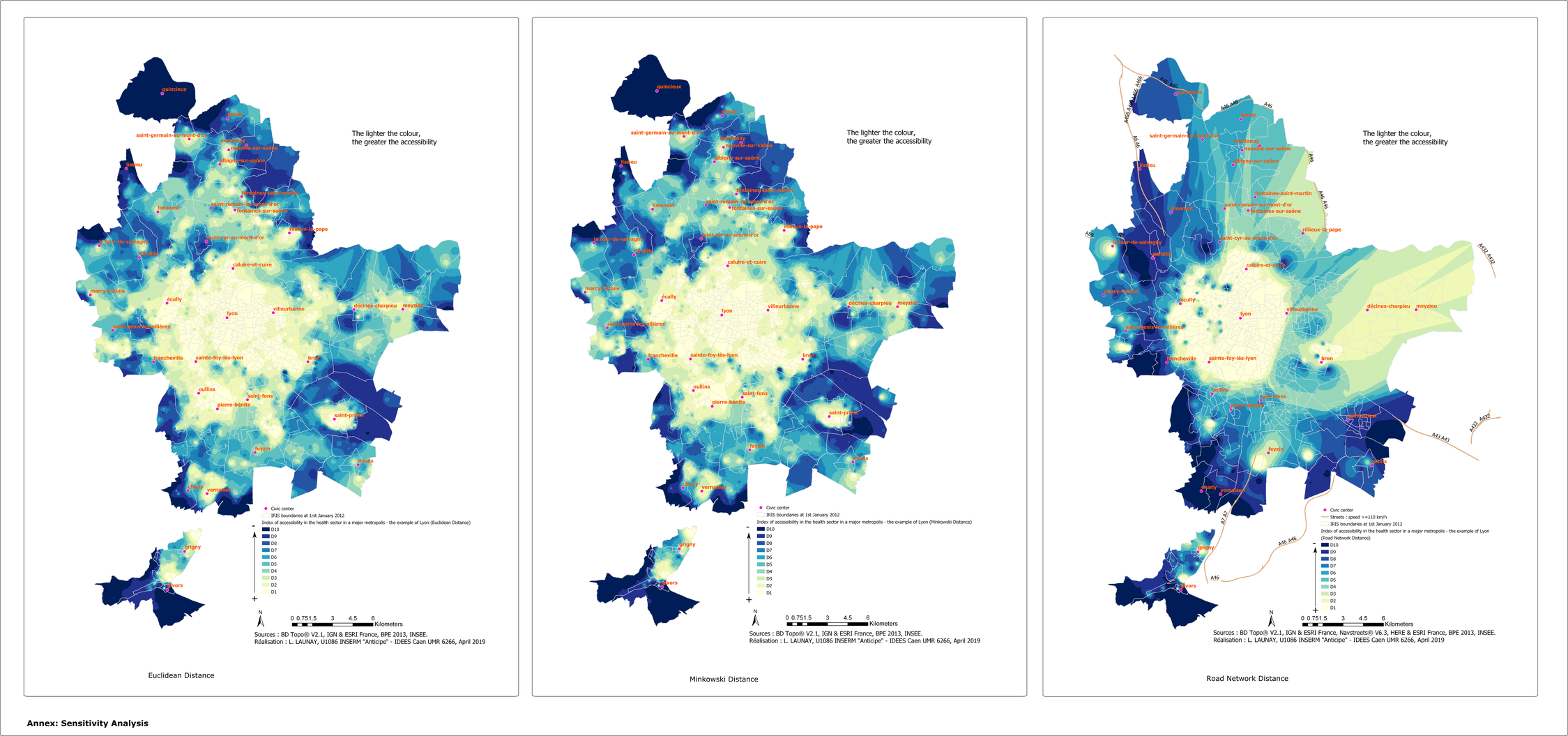

Supplement: S1 Fig — (TIF) [file pone.0221417.s004.tif]
